# Quantitative Trait Locus Mapping and Candidate Gene Analysis for *Verticillium* Wilt Resistance Using *Gossypium barbadense* Chromosomal Segment Introgressed Line

**DOI:** 10.3389/fpls.2018.00682

**Published:** 2018-05-30

**Authors:** Jun Zhao, Jianguang Liu, Jianwen Xu, Liang Zhao, Qiaojuan Wu, Songhua Xiao

**Affiliations:** ^1^Key Laboratory of Cotton and Rapeseed, Ministry of Agriculture, Institute of Industrial Crops, Jiangsu Academy of Agricultural Sciences, Nanjing, China; ^2^Provincial Key Laboratory of Agrobiology, Jiangsu Academy of Agricultural Sciences, Nanjing, China

**Keywords:** cotton, *Verticillium* wilt, CSIL, quantitative trait loci (QTLs), resistance gene

## Abstract

*Verticillium* wilt (VW) is a soil-borne fungal disease that is caused by *Verticillium dahliae* Kleb and seriously damages cotton production annually in China. To date, many efforts have been made to improve the resistance of upland cotton against VW, but little progress has been achieved because of a lack of resistant upland cotton to VW. *G. barbadense* is known to carry high resistance to VW; however, it is difficult to transfer the resistance trait from *G. barbadense* to upland cotton because of linkage drag and distortion in the interspecific hybrid. In this study, a chromosomal segment introgression line (CSIL), SuVR043, containing a single and homozygous chromosome segment of *G. barbadense* cv. H7124 D04 (Chr 22), was created and used to construct an F_2_ population for mapping of VW resistance quantitative trait loci (QTLs) in the greenhouse. Two major resistance QTLs against nondefoliating *V. dahliae* isolate Bp2, called qVW-Bp2-1 and qVW-Bp2-2, which were flanked by the markers cgr6409-ZHX37 and ZHX57-ZHX70 and explained an average of 16.38 and 22.36% of the observed phenotypic variation, respectively, were detected in three independent replicate experiments. The genetic distances from cgr6409 to ZHX37 and from ZHX57 to ZHX70 were 2.4 and 0.8 cM, respectively. By analyzing the genome sequence of the qVW-Bp2-1 and qVW-Bp2-2 regions, we determined that the accurate physical distances from cgr6409 to ZHX37 and from ZHX57 to ZHX70 in the *G. barbadense* genome are 254 and 140 kb, and that those spans 36 and 20 putative genes, respectively. The results of the expression analysis showed significant differences in the expression profiles of *GbCYP450, GbTMEM214*, and *GbRLK* among *G. barbadense* cv. H7124, CSIL SuVR043 and *G. hirsutum* acc. Sumian 8 at different times after inoculation with *V. dahliae* isolate Bp2. Virus-induced gene silencing (VIGS) analysis showed that silencing of *GbCYP450* and *GbTMEM214* decreased H7124 and CSIL SuVR043 resistance to VW. These results form a solid foundation for fine mapping and cloning of resistance genes in the substituted segment and will provide valuable assistance in future efforts to breed for VW resistance.

## Introduction

Cotton (*Gossypium* spp.) is widely cultivated for the important economic value of its fiber. Verticillium wilt (VW), caused by *Verticillium dahliae* (*V. dahliae*), is the major limiting factor of cotton producers and leads to yield loss every year. *V. dahliae* is a soil-borne pathogen of vascular plants and has a broad host range (over 200 dicotyledonous plant species) (Fradin and Thomma, 2006), different strains vary in virulence against different hosts. A great deal of research in comparative genomics has revealed that the *V. dahliae* can degrade plant cell walls by the expansion of some CAZyme families, and that flexible genomic islands containing TE and LS genes contribute to the development of virulence and host adaptation by the De et al. (2013). In China, *V. dahliae* strains that have cotton as their main host are divided into defoliating and nondefoliating strains. The main differences between them are that the defoliating strains develop into epidemics earlier and more rapidly and cause significantly greater yield losses than the nondefoliating strains at equal inoculum densities (BejaranoAlcazar et al., 1995). In addition, the defoliating strains can cause to be completely defoliated, whereas the nondefoliating strains cause only chlorosis and mild wilting of leaves, not defoliation (Schnathorst and Mathre, 1966). The recent nation-wide distribution of large quantities of cotton seed without implementation of proper quarantine procedures before distribution may have contributed to the widespread dissemination of the defoliating type in major cotton cultivating areas (Xia et al., 1998). VW is particularly difficult to prevent in cotton, and no efficient method has been developed to control this disease. Therefore, breeding and planting resistant varieties to VW is the most effective and economical method to control the epidemic. Along with a lack of immune or highly resistant germplasm, the genetic and molecular mechanisms of cotton resistance to *V. dahliae* infection are poorly understood, and conventional breeding for VW resistance has not been successful (Cai et al., 2009). Creating VW-resistant cotton germplasm through genetic engineering will be the main research objective in the future. The fine mapping and cloning of resistant genes will be the key to achieve this goal.

The resistance mechanism of cotton to VW is very complicated and involves a number of signaling pathways and biochemical substances (Fradin and Thomma, 2006). Previous studies have revealed that a series of basal defense responses and accumulated phytoalexin, including terpenoids and phenylpropanoid, play an important role in the response of cotton to VW infection. In addition, lignin and lignin-like phenolic polymers also serve important functions (Mace et al., 1990; Gayoso et al., 2010; Xu et al., 2011). Signaling transduction pathways are known to play central roles in the perception of attacks and activation of plant defense responses. To date, some results have indicated that reactive oxygen species, salicylic acid (SA), jasmonic acid (JA), ethylene (ET), brassinosteroids (BRs), spermine, and camalexin take part in the resistance of cotton to VW (Johansson et al., 2006; Gao W. et al., 2013; Mo et al., 2015; Guo et al., 2016). To reveal the resistance mechanism of cotton against VW, researchers have studied a number of related gene, *Ve1* in tomato is the most famous such gene and endows some tomato varieties with high VW resistance (Kawchuk et al., 2001; Fradin et al., 2009, 2011); its resistance mechanism has been investigated by many researchers (Fradin et al., 2009; Gayoso et al., 2010; Jonge et al., 2012; Zhang Z. et al., 2013). However, this gene does not improve the resistance of cotton to *V. dahliae* (Liu et al., 2014; Song et al., 2017). A series of homologous *Ve* genes in cotton have also been identified, but no reports have shown they were successfully used in cotton breeding (Zhang et al., 2011; Zhang J. F. et al., 2012).

In addition to the *Ve* gene, several other genes have been reported to improve resistance to VW, including *GbTLP1* (Munis et al., 2010), *GbCAD1, GbSSI2* (Gao W. et al., 2013), *GbSTK* (Zhang Y. et al., 2013), *GbWRKY1* (Li et al., 2014), *GbRLK* (Zhao et al., 2015), *GhPAO* (Mo et al., 2015), *Gbvdr5* (Yang Y. W. et al., 2015), *GbERF1-like* (Guo et al., 2016), *GhSAMDC* (Mo et al., 2016), *GbNRX1* (Li et al., 2016), *GhLMM* (Chai et al., 2017), and more. Additionally, some exogenous genes can also improve resistance to VW in transgenic cotton, including *GAFPs* (Wang et al., 2016), *Hpa1*_*Xoo*_ (Miao et al., 2010), *NaD1* (Gaspar et al., 2014), *p35* (Tian et al., 2010), and *Hcm1* (Zhang et al., 2016). In contrast to the traditional hybridization-based approaches, high-throughput sequencing technologies, referred to as RNA-Seq and proteomics, provide a large amount of information regarding the resistance mechanisms of cotton against VW (Xu et al., 2011; Gao W. et al., 2013). The *Agrobacterium*-mediated VIGS assay is another method for analyzing genes that confer resistance to VW. To data, several genes have been shown to participate in VW resistance, including *GhNDR1, GhMKK2*, and *GhBAK1* (Gao et al., 2011; Gao X. et al., 2013).

With respect to the inheritance of resistance and improving genetic resistance to VW, researchers have carried out many studies on the loci encoding disease resistance traits by using different materials, different genetic groups or different identifying environments (Yang et al., 2008; Ning et al., 2013; Wang et al., 2014; Zhang J. F. et al., 2015; Shi et al., 2016; Palanga et al., 2017). To date, many VW resistance quantitative trait loci (QTLs) have been reported, and most of them have been detected in *G. barbadense* × *G. hirsutum* populations (Zhang J. F. et al., 2015). *G. hirsutum* is the most widely planted cotton species in China and has been used as the subject of most genetic studies and breeding efforts. It accounts for more than 95% of annual cotton production worldwide (National Cotton Council, http://www.cotton.org/, 2006), but most commercial cultivars of the species are susceptible or only tolerant to VW (Cai et al., 2009). *G. barbadense* is characterized by extra-long-staple cotton compared to *G. hirsutum*. Many *G. barbadense* genotypes are known to possess a high level of resistance to VW (Wilhelm et al., 1974; Zhang J. F. et al., 2012, 2015; Zhou et al., 2014). Hence, cotton breeders have made great efforts to introgress the resistance genetic fragment(s) or gene(s) from *G. barbadense* to *G. hirsutum*, with the expectation of breeding new commercial cotton cultivars with both high yield and high VW resistance. However, most of these resistance fragment(s) or gene(s) have not been successfully transferred into commercial upland cotton due to the linkage drag between resistance and undesired agronomic traits, as well as distortion in segregation of the interspecific hybrids (Cai et al., 2009; Zhang J. F. et al., 2015).

To order to break down the linkage drag and distortion in interspecific hybrids, a set of chromosomal segment introgression lines (CSILs) have been developed by crossing *G. hirsutum* and *G. barbadense* (Wang et al., 2008, 2014; Zhai et al., 2016). Because CSILs carry introgressed chromosomal segments on the same genetic background, these lines provide the ideal material for studying the genetic functions of chromosomal segments, particularly for QTL mapping. To data, fine mapping of QTLs using CSILs has been reported in different plants, including tomato, rape, rice, and cotton (Zamir, 2001). In this study, *G. hirsutum* acc. Sumian 8 was used as the recipient parent and *G. barbadense* cv. H7124 as the donor. By marker-assisted selection (MAS), we created the highly VW-resistant stain CSIL SuVR043, featuring the introgression of *G. barbadense* cv. H7124 chromosome 22 (D04). For mapping of the VW resistance QTLs in this region, we developed a population of 1,100 individuals and constructed a linkage group with an average genetic distance of 0.35 cM between loci in this study; two major resistance QTLs for *V. dahliae* nondefoliating isolate Bp2, qVW-Bp2-1, and qVW-Bp2-2, were detected on the introgressed chromosome segment in three independently replicated experiments. The candidate genes in the QTL regions were selected according to analysis of gene sequence divergences, screening of gene expression patterns and VIGS analysis. The results of this study establish a foundation for fine mapping and cloning of the resistance gene in the future.

## Materials and methods

### Plant materials and development of cotton CSIL SuVR043

**Figure 1:**
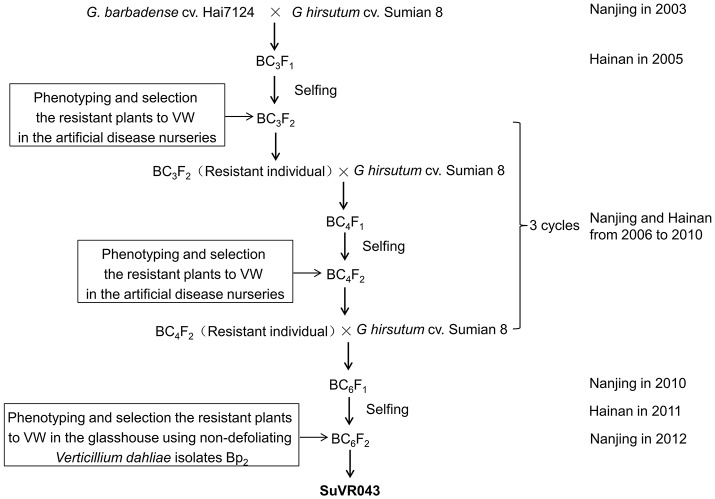
Breeding scheme for development of *G. hirsutum* cv. CSIL SuVR043.

*G. hirsutum* cv. Sumian 8 was bred by the cotton foundation seed farm in Taicang, China, and released by the Jiangsu Crops Variety Approval Committee in 1988; this cultivar is susceptible to VW. *G. barbadense* cv. H7124, grown extensively and serving as a widely used VW-resistant cultivar for genetic study and breeding in China, is the offspring of a selected individual in studies of the inheritance of resistance to *V. dahliae*. The creation scheme for CSIL SuVR043 is described in Figure 1. The BC_3_F_1_ was generated by 3 cycles of crossing using Sumian 8 as the recipient and H7124 as the donor parent, conducted from 2003 to 2005 at the Cotton Breeding Station of Jiangsu Academy of Agricultural Sciences; BC_3_F_1_ plants were grown at Hainan Plant Experiment Station in the winter of 2005 and were self-pollinated to produce the BC_3_F_2_ generation. The BC_3_F_2_ generation was planted in the artificial disease nurseries in Nanjing, and then resistant plants were selected and backcrossed using Sumian 8 as the male parent. In the winter of the same year, the hybrids were grown at Hainan Plant Experiment Station and self-pollinated to produce the generation for disease resistance identification in the artificial disease nurseries of Nanjing in the following year. Continuous identification, backcrossing and selfing were conducted for 3 cycles to acquire the BC_6_F_1_ generation from 2006 to 2010. In the winter of 2012, the BC_6_F_2_ generation grown in a greenhouse was inoculated with the nondefoliating *V. dahliae* isolate Bp2 and 7 resistant plants were selected. Meanwhile, the foreground and background of these 7 resistant plants were selected using 470 simple sequence repeat (SSR) markers distributed on 26 cotton chromosomes based on *G. hirsutum*×*G. barbadense* maps (Zhao et al., 2012), and one plant was identified to contain a single and homozygous *G. barbadense* introgressed chromosome segment; this plant was named SuVR043 (Supplementary Figure 1), and its resistance was higher significantly than that of Sumian 8 in the three subsequent VW resistance evaluation experiments. In 2014, SuVR043 and Sumian 8 were crossed in Nanjing, and 58 F_1_ seeds were planted and self-pollinated in Hainan to produce F_2_ progeny. In 2015, the F_2_ population, containing 1,100 individuals, was planted in Nanjing and self-pollinated to produce F_2:3_ family seeds.

### VW resistance evaluation under greenhouse conditions

Resistance evaluation under greenhouse conditions was also conducted in three independently repeated experiments from October 2015 to April 2016 using the Chinese national standard method. Three independently repeated experiments were performed in three different greenhouses at the same time. In every experiment, the parents and F_2:3_ family lines were planted in a completely randomized design with three replications. For each replicate, the disease grades of at least 15 plants were counted for every F_2:3_ family line. Sulfuric-acid-delinted seeds of each F_2:3_ line and the parents lines were germinated in paper cups (250 mL) containing a soil mixture of vermiculite and peat (2:1 volume ratio) in three greenhouses. The temperature of the greenhouses was controlled to approximately 26°C in the daytime and 22°C at night. When cotton seedlings developed two true leaves stage, the bottom of the paper cup was torn out, and the cup was placed into a new paper cup with 20 ml conidial suspensions (2 × 10^7^ conidia/ml), after which the seedlings were kept at a similar temperature and a high humidity environment. Thirty-five days after inoculation, disease symptoms were scored as described in a previous report (Ning et al., 2013). The disease grades of 0, 1, 2, 3, and 4 for leaf disease symptoms were defined as follows: 0 indicates a healthy plant with no disease symptoms; 1 indicates ≤25.0% of leaves exhibited disease symptoms; 2 indicates 25.1–50.0% of leaves exhibiting disease symptoms; 3 indicates 50.1–75.0% of leaves exhibiting disease symptoms; 4 indicates >75.0% of leaf surfaces exhibiting disease symptoms, with plants completely defoliated or dead. The VW disease index was calculated using the following formula:

$$DI\left(\%\right)=\frac{\overset{n}{\underset{i=1}{\sum }}XiYi}{4N}\times 100$$

The DI is the VW disease index of each F_2:3_ family, X is the disease grade (from 0 to 4), Y is the number of plants with the corresponding disease grade, and N is the total number of investigated plants from each F_2:3_ family.

In this study, the nondefoliating *V. dahliae* isolate Bp2 was used as a representative of medium virulence varieties in the Yangtze River cotton-growing region. The *V. dahliae* isolate was grown on solid potato sucrose agar culture medium at room temperature (25°C) for 7 d, then inoculated into liquid potato sucrose culture medium and oscillated for 7 d, filtered using 2-layers of gauze and diluted to a final concentration of 2 × 10^7^ conidia/ml.

### *V. dahliae* biomass quantification in plants

CSIL SuVR043, Sumian 8 and H7124 were inoculated with *V. dahliae* isolate Bp2 when they had developed two true leaves, as described above. At 21 d after inoculation, the lower 2 cm of the stems of CSIL SuVR043, Sumian 8, and H7124 were harvested from at least 20 plants and flash frozen in liquid nitrogen. The samples were mixed and ground to powder, approximately 100 mg of which was used for DNA isolation. Quantitative reverse-transcriptase polymerase chain reaction (qRT-PCR) was conducted with an ABI PRISM 7500 PCR machine (Applied Biosystems, USA) with the SYBR Premix Ex Taq II kit (TaKaRa, Japan, Code No. RR820A). For *V. dahliae* biomass measurement, the internal transcribed spacer region of the ribosomal DNA was targeted to generate a 200 bp amplicon using the fungus-specific primer ITS1-F (5-AAAGTTTTAATGGTTCGCTAAGA-3) (Gardes and Bruns, 1993) and the *V. dahliae*-specific reverse primer ST-VE1-R (5-CTTGGTCATTTAGAGGAAGTAA-3) (Lievens et al., 2006). *EF-1*α (Acc.No.AJ223969) from cotton was used as an internal control for normalization of the different DNA samples.

### DNA extraction, development of SSR primer pairs and molecular marker analysis

DNA was extracted from leaf samples of 1100 F_2_ plants using the CTAB extraction procedure as described by Paterson et al. (1993). Some of the microsatellite primer pairs used in this experiment are available in the Cotton Marker Database (CMD) (https://www.cottongen.org). Based on the selected result of the foreground and background of CSIL SuVR043, we extracted the sequence of the introgression segment and designed SSR primer pairs using the recently released cotton D genome sequence as a reference. The SSR loci were identified and characterized using the program SSR locator (da Maia et al., 2008). Forward and reverse flanking primer pairs were designed using Primer 3 (Rozen and Skaletsky, 2000). The details of the SSR primers development strategy were as described by Lu et al. (2010). PCR amplifications were conducted on an Eastwin PCR machine (ETC811) and performed according to the method described in Zhang et al. (2000); the PCR products were electrophoresed on a 10 % non-denaturing polyacrylamide gel. DNA fragments were visualized by staining with silver nitrate, following the procedure of Zhang et al. (2000).

### QTL analysis

The genetic linkage map and orientation of the SSR markers were determined using the software JoinMap version 3.0 with a logarithm of the odds (LOD) value of 3.0 (Ooijen and Voorrips, 2001). The conversion of recombination frequencies into centimorgans (cM) was calculated with the Kosambi map function (Kosambi, 2016). The graphic representation of the linkage groups was created using MapChart 2.2 (Voorrips, 2002). QTLs were identified by composite interval mapping (Zeng, 1994) using Windows QTL Cartographer 2.5 (Wang et al., 2012), and an LOD threshold of 2.5 was used in the F_2_ population.

### Gene prediction and annotation in the target region

The linkage markers with QTLs were anchored to the cotton reference genome sequence of the tetraploid *G*. *hirsutum* and *G*. *barbadense* with alignments of E-value < le-10 using BLASTN (Altschul et al., 1990). The sequences were first extracted from the tetraploid *G*. *hirsutum* and *G*. *barbadense* and were then uploaded into a local Blast2Go system (Conesa et al., 2005) to perform functional annotation.

### RNA extraction and candidate gene expression analysis qRT-PCR

Seedlings of H7124, Sumian 8, and CSIL SuVR043 displaying two true leaves stage were inoculated with *V. dahliae* spore suspension, and mock-inoculated plants dipped in sterile water were used as the control. The roots of samples were harvested at time intervals of 0, 24, 48, 96, and 144 h from inoculation and were immediately frozen in liquid nitrogen for subsequent determination of expression responses. Total RNA was extracted from cotton root samples using an E.Z.N.A^TM^ Plant RNA Kit (Omega, Norcross, GA, USA; cat: R6827-01). First-strand cDNA was synthesized in a final reaction volume of 20 μl containing 2 μg of RNA according to the instructions of the PrimeScript™ RT Reagent Kit with gDNA Eraser Kit (TaKaRa, Japan, Code No. RR047A). The synthesized cDNAs were utilized as templates in the following qRT-PCR reactions. All qRT-PCR reactions were performed on an ABI PRISM 7500 Real-Time PCR System using the SYBR Premix Ex Taq II kit (TaKaRa, Japan, Code No. RR820A) according to the manufacturer's instructions. Three biological replicates were conducted for each qRT-PCR reaction. The cotton *Histone* 3 gene (accession number: AF024716) was used as an internal reference. The relative expression level was calculated as 2^−ΔΔΔ^CT; ΔΔΔCT = [(Ct_gene_−Ct_His_)_xh_–(Ct_gene_−Ct_His_)_0h_]_V.D_−[(Ct_gene_−Ct_His_)_xh_−(Ct_gene_−Ct_His_)_0h_]_CK_. qRT-PCR specific primers are listed in Supplementary Table 1 and were designed using Beacon Designer 7.0 from Premier Biosoft International, Palo Alto, CA.

### VIGS in cotton followed by *V. dahliae* inoculation

The fragments of Cytochrome p450 78a5-like (*GbCYP450*), Transmembrane protein 214a (*GbTMEM214*) and Receptor-like protein kinase (*GbRLK*) genes were amplified by PCR using primer pairs with the restriction sites for tobacco rattle virus (TRV) vectors shown in Supplementary Table 2 from the cDNA of *G. barbadense* cv. H7124 and were digested with the corresponding restriction enzyme and then ligated into the TRV:00 plasmid to generate pTRV2:GbCYP450, pTRV2:GbTMEM214, and pTRV2:GbRLK. pTRV2:GbCLA1 (Cloroplastos alterados 1), causing an albino plant phenotype was used as a positive control. These constructs and pTRV1 were transformed into *Agrobacterium tumefaciens* strain GV3101 by electroporation. *Agrobacterium* cultures containing pTRV2:Target gene and pTRV1 were grown overnight at 28°C in liquid LB medium containing 50 mg/L kanamycin and 25 mg/L rifampicin to an OD of approximately 0.8. The cells were pelleted by centrifugation at 1,180 g at room temperature for 5 min and resuspended in an infiltration culture containing 10 mM MgCl_2_, 10 mM MES and 200 μM acetosyringone. Cell suspensions containing pTRV2:Target gene and pTRV1 were incubated at room temperature for at least 3 h and then mixed at a 1:1 ratio and infiltrated into the two fully expanded cotyledons of *G. barbadense* cv. Hai 7124 and CSIL SuVR043 seedlings. The treated seedlings were then grown in a controlled-environment chamber at 25°C with a 16/8 h light/dark photoperiod cycle and a relative humidity of 80%. When the albefaction phenotype of the leaves occurred 2 weeks after infiltration with pTRV2:GbCLA1, the expression of target genes in all plantlets treated by VIGS was checked by qRT-PCR with specific primers. Then, the positive plantlets with silent target genes were inoculated with *V. dahliae* as previously described in this paper. The disease index was calculated from 20 plants per treatment in each of three repetitions. The standard of VW resistance evaluation for each treatment was evaluated as described previously in this paper.

## Results

### Genome-wide analysis of *G. hirsutum* cv. CSIL SuVR043 with SSR markers

In a previous study, 470 SSR markers that were approximately evenly spaced across the cotton genome based on *G. hirsutum* × *G. barbadense* maps were used to analyze the foreground and background of CSIL SuVR043 (Zhao et al., 2012), and this study detected one SSR marker (NAU3392) that was polymorphic between *G. hirsutum* cv. Sumian 8 and CSIL SuVR043. We constructed the F_2_ population containing 176 individuals of CSIL SuVR043 × Sumian 8. Based on the F_2:3_ phenotypes of the resistance to *V. dahliae* strain Bp2, the result of the single-marker analysis showed the resistance locus was linked to marker NAU3392 with a *p* value of 2.3 × 10^−12^. Subsequently, we used 3100 SSR markers to further select the background of CSIL SuVR043, of which 1,750 markers were mapped and distributed on 26 cotton chromosomes based on the published genetic map of *G. hirsutum* × *G. barbadense* (Zhao et al., 2012), and identified four makers (NAU3392, NAU3791, NAU5294, and JESPR220) to be polymorphic between CSIL SuVR043 and Sumian 8. Based on the *G. hirsutum* × *G. barbadense* maps, these four makers (NAU3392, NAU3791, NAU5294, and JESPR220) were mapped and were found to be distributed on Chr 22 (D04) of the cotton genome. In 2013, we selected all 86 SSR markers of Chr 22 group based on *G. hirsutum* × *G. barbadense* maps (Zhao et al., 2012) to analyze the background of CSIL SuVR043 and identified that eight makers (NAU3392, NAU6992, NAU6993, NAU3791, cgr6409, NAU5294, JESPR220, and NAU7290) showed identical banding patterns between *G. barbadense* L. cv. H 7124 and CSIL SuVR043, in contrast to the polymorphic patterns between CSIL SuVR043 and *G. hirsutum* L. cv. Sumian 8, and 78 other SSR markers like NAU476 and NAU2291 showed identical banding patterns between *G. hirsutum* L. cv. Sumian 8 and CSIL SuVR043 (Supplementary Figure 1, Supplementary Table 3). Then, through the continuous two self-generations and the marker selection, we ensured the introgressed segment to be homozygous. The above results showed that CSIL SuVR043 contained a single and homozygous chromosome introgressed segment of *G. barbadense* cv. H7124 Chr 22 (D04).

### Comparison of agronomic characters and vw resistance performance between Sumian8 and CSIL SuVR043

**Figure 2:**
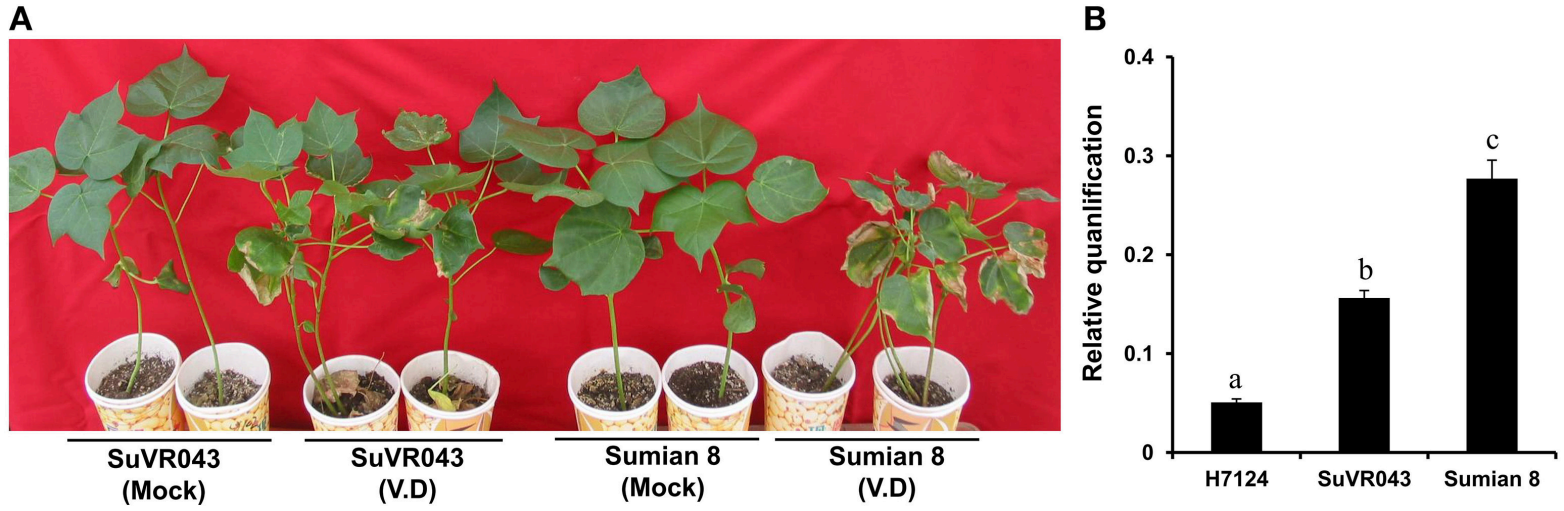
Resistance phenotypes and detection of *V. dahliae* biomass of two parents, CSILSuVR043 and Sumian 8, after artificial VW inoculation in 2014 in Nanjing. **(A)** The resistance phenotype of CSILSuVR043 and Sumian 8. The picture was taken 4 weeks after inoculation. **(B)** Detection of *V. dahliae* biomass in CSILSuVR043, Sumian 8 and H7124 using qRT-PCR. DNA was extracted from the lower 2 cm of stems 21 days after inoculation with *V. dahliae*. The relative average fungal biomass is shown with standard errors. The letter indicates significant differences according to Duncan's multiple range tests (*p* < 0.05).

To learn more about the characteristics of CSIL SuVR043, we compared the main agronomic traits, fiber quality and disease resistance of CSIL SuVR043, Sumian 8 and H7124. The results showed that there were no significant differences in main agronomic traits and fiber quality except for VW resistance between CSIL SuVR043 and Sumian 8 (Figure 2A, Table 1). This indicated that the chromosome introgressed segment from *G. barbadense* cv. H7124 Chr 22 (D04) significantly increased CSIL SuVR043 resistance to VW. Meanwhile, we detected the biomass of *V. dahliae* in CSIL SuVR043, Sumian 8, and H7124 after 35 days inoculation using qRT-PCR. The results showed that the proliferation of *V. dahliae* in Sumian 8 was significantly higher than that in CSIL SuVR043 and H7124. This suggested that CSIL SuVR043 and H7124 could inhibit the spread of *V. dahliae in vivo* compared to Sumian 8 (Figure 2B).

**Table 1:** CSILSuVR043, Sumian 8 and H7124 mean values for agronomic and fiber traits and resistance to VW.

**Characters**	**Sumian 8**	**SuVR043**	**H 7124**
Plant height (cm)	98.3 ± 3.1	99.5 ± 2.2	119.3 ± 6.8^*^
Number of fruit branches	15 ± 1.6	14.8 ± 2.9	17 ± 3.0^*^
Boll number per plant	28.36 ± 2.2	28.12 ± 2.1	25 ± 3.0^*^
Single boll weight (g)	5.3 ± 0.6	5.1 ± 0.6	3 ± 0.5^**^
Lint percent (%)	39.8 ± 0.9	39.2 ± 1.1	35.1 ± 0.8^**^
Seed cotton yield (kg)	261.8 ± 16.2	258.6 ± 11.9	65.4 ± 6.9^**^
Lint yield (kg)	102.2 ± 9.3	100.6 ± 8.1	21.2 ± 3.1^**^
Seed index(g)	10.1 ± 0.9	9.9 ± 1.3	10.2 ± 1.1
Lint index(g)	7 ± 0.5	6.9 ± 0.4	5.1 ± 0.5^**^
Fiber length (mm)	28.2 ± 3.2	28.1 ± 2.8	32.8 ± 3.2^**^
Fiber uniformity (%)	84.5 ± 6.3	84.2 ± 4.9	86.8 ± 5.0^*^
Micronaire value	5.4 ± 0.6	5.5 ± 0.3	3.9 ± 0.3^**^
Fiber elongation (%)	5.2 ± 0.8	5.3 ± 0.7	7.2 ± 0.5^**^
Fiber strength (cN·tex^−1^)	31.5 ± 2.6	31.2 ± 1.9	35.3 ± 3.3^**^
Disease index (%)	59.8 ± 7.9	23.4 ± 4.1^**^	9.0 ± 1.6^**^

CSIL SuVR043, Sumian 8 and H7124 were grown on a farm at the Cotton Breeding Station of Jiangsu Academy of Agricultural Sciences, China in the 2014 cotton-growing seasons. Seeds were planted in a field without diseased soil. Data represent the mean ± SE (n ≥ 15); similar results were obtained from three independent experiments.

* indicates significance at p < 0.05,

** *indicates significance at p < 0.01. Disease index represents the results 6 weeks after inoculation*.

### Development of SSR primer pairs and construction of a genetic map

Based on *G. hirsutum* × *G. barbadense* maps (Zhao et al., 2012), the introgressed chromosome segment was flanked by NAU476 and NAU2291. To further fine map the VW resistance QTL in the introgressed chromosome segment from *G. barbadense* cv. H7124, we searched the sequence of 10 markers (NAU476, NAU3392, NAU6992, NAU6993, NAU3791, cgr6409, NAU5294 JESPR220, NAU7290, and NAU2291) against the complete genome sequence of *G. raimondii*. Among them, the markers NAU476 and NAU2291 were adjacent to NAU3392 and NAU7290, respectively, and were not polymorphic between CSIL SuVR043 and Sumian 8. The search result showed that the forward and reverse sequences of seven markers (NAU6992, NAU6993, NAU3791, cgr6409, JESPR220, NAU7290, and NAU2291) were located in the genome, but the reverse sequence of marker NAU3392, the forward sequence of marker NAU476 and the reverse sequence for marker NAU5294 were not (Supplementary Table 4). We extracted the sequence from the reverse sequence of marker NAU2291 to the reverse sequence of the marker NAU476 in *G. raimondii* genome (32304099–35343399) and developed 189 SSR primer pairs (Supplementary Table 5). Among them, 42 SSR markers showed polymorphisms between CSIL SuVR043 and Sumian 8. Using 49 SSRs showing polymorphisms, we genotyped 1,100 individuals and constructed a linkage group using MapChart 2.2. The group included 49 markers and 32 loci and spanned 11.1 cM. There were 8 loci containing at least 2 co-segregating markers.

### Phenotypic evaluations of the parents and F_2_ population resistance to VW in greenhouse investigations

**Figure 3:**
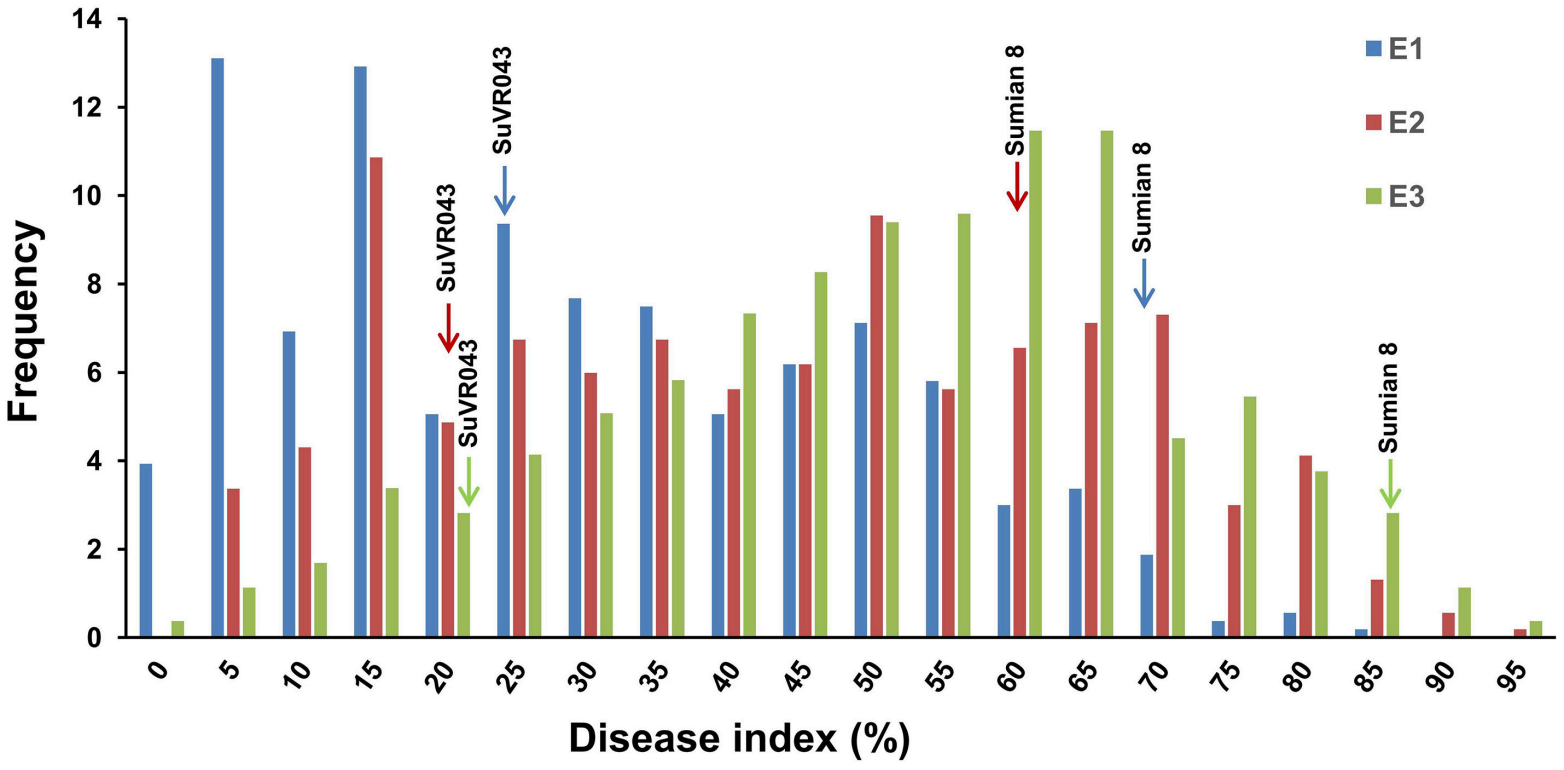
Frequency distributions of disease index for three independently repeated VW resistance evaluation experiments in the greenhouse. E1, E2, and E3 denote three VW resistance evaluation experiments that were performed from October 2015 to April 2016 in three different greenhouses.

VW resistance evaluation experiments under greenhouse conditions were performed from October 2015 to April 2016 in three different greenhouses. The plant disease response in both parental lines and F_2:3_ family lines were evaluated in terms of leaf symptoms under greenhouse conditions, and the distribution of different resistance levels frequencies of the parents and F_2:3_ family lines are shown in Figure 3, Table 2. The evaluations of VW resistance for the F_2:3_ family lines were performed three times in the greenhouse using *V. dahliae* isolate Bp2. The DI values of the F_2:3_ family lines varied from 0.00 to 89.71% in the first experiment, from 2.6 to 100% in the second experiment, and from 0.00 to 100% in the third experiment. The broad-sense heritabilities were 0.69, 0.64, and 0.72 in the three independent repeated experiments, respectively. The mean DI value of the F_2:3_ family lines was 25.32% in the first experiment, which was close to that of the resistant parent SuVR043 (20.24%), and in the second and third experiments, the mean DI values were 43.03 and 50.86%, respectively, which were close to that of the midparent (40.53 and 54.29%).

**Table 2:** Descriptive statistics and broad-sense heritability (H_2_) of disease index in greenhouse tests.

**Test**	**F**_**2**_ **population**						**Parent**			**H 7124**	**H_2_**
	**Mean**	**Min**	**Max**	**SD**	**Skewness**	**Kurtosis**	**SuVR043**	**Sumian 8**	**Midparent**		
E1	25.32	0.00	89.71	20.25	0.48	−0.64	20.24	71.88	46.06	6.67	0.69
E2	43.03	2.60	100.00	22.77	0.14	−0.98	16.47	64.58	40.53	4.33	0.64
E3	50.86	0.00	100.00	20.55	−0.09	−0.35	19.79	88.79	54.29	2.50	0.72

*E1, E2, and E3 represent three independently repeated VW resistance evaluation experiments that were performed from October 2015 to April 2016 in three different greenhouses. SD, standard deviation*.

### QTL detection of VW resistance by composite interval mapping

Three VW resistance QTLs were detected on the introgressed chromosome segment using *V. dahliae* nondefoliating isolate Bp2 as shown in Table 3. The QTLs qVW-Bp2-1 and qVW-Bp2-2 flanked by the markers cgr6409-ZHX37 and ZHX57-ZHX70 were detected in three independent repeated experiments, with average explained phenotypic variances of 16.38 and 22.36% and LOD scores ranging from 3.2 to 8.8 and 4.9 to 9.6, respectively. The QTL qVW-Bp2-3 flanked by the markers ZHX102 and NAU5294 was detected only in one experiment with an average explained phenotypic variance of 5.1% and an LOD value of 4.7. Taken together, the results showed that there are two major resistance QTLs for *V. dahliae* nondefoliating isolate Bp2 on the introgressed chromosome segment.

**Table 3:** QTL analyses for VW resistance detected by composite interval mapping in the F_2_ generations under greenhouse conditions.

**Test**	**QTL**	**Position (cM)**	**Flanking marker**	**LOD value**	**Additive**	**Dominance**	***r^2^*(%)**	**Origin**
E1	qVW-BP_2_-1	7.7	Cgr6409-ZHX30	8.8548	−0.0693	0.0945	16.36	SuVR043
	qVW-BP_2_-2	9.7	ZHX57-ZXH70	9.5925	−0.1603	0.1238	24.61	SuVR043
E2	qVW-BP_2_-1	7.7	Cgr6409-ZHX30	5.5789	−0.0698	0.0827	16.51	SuVR043
	qVW-BP_2_-3	0.3	ZHX102-NAU5294	4.6797	−0.0732	0.1039	5.1	SuVR043
	qVW-BP_2_-2	9.7	ZHX57-ZXH70	7.5143	−0.1526	0.1527	21.42	SuVR043
E3	qVW-BP_2_-1	7.7	Cgr6409-ZHX30	3.2347	−0.0698	0.1143	15.98	SuVR043
	qVW-BP_2_-2	9.7	ZHX57-ZXH70	4.8602	−0.1433	−0.0318	21.06	SuVR043

*E1, E2, and E3 represent three independently repeated VW resistance evaluation experiments that were performed from October 2015 to April 2016 in the three different greenhouses*.

### Prediction of candidate genes relevant to the VW resistance QTL

**Figure 4:**
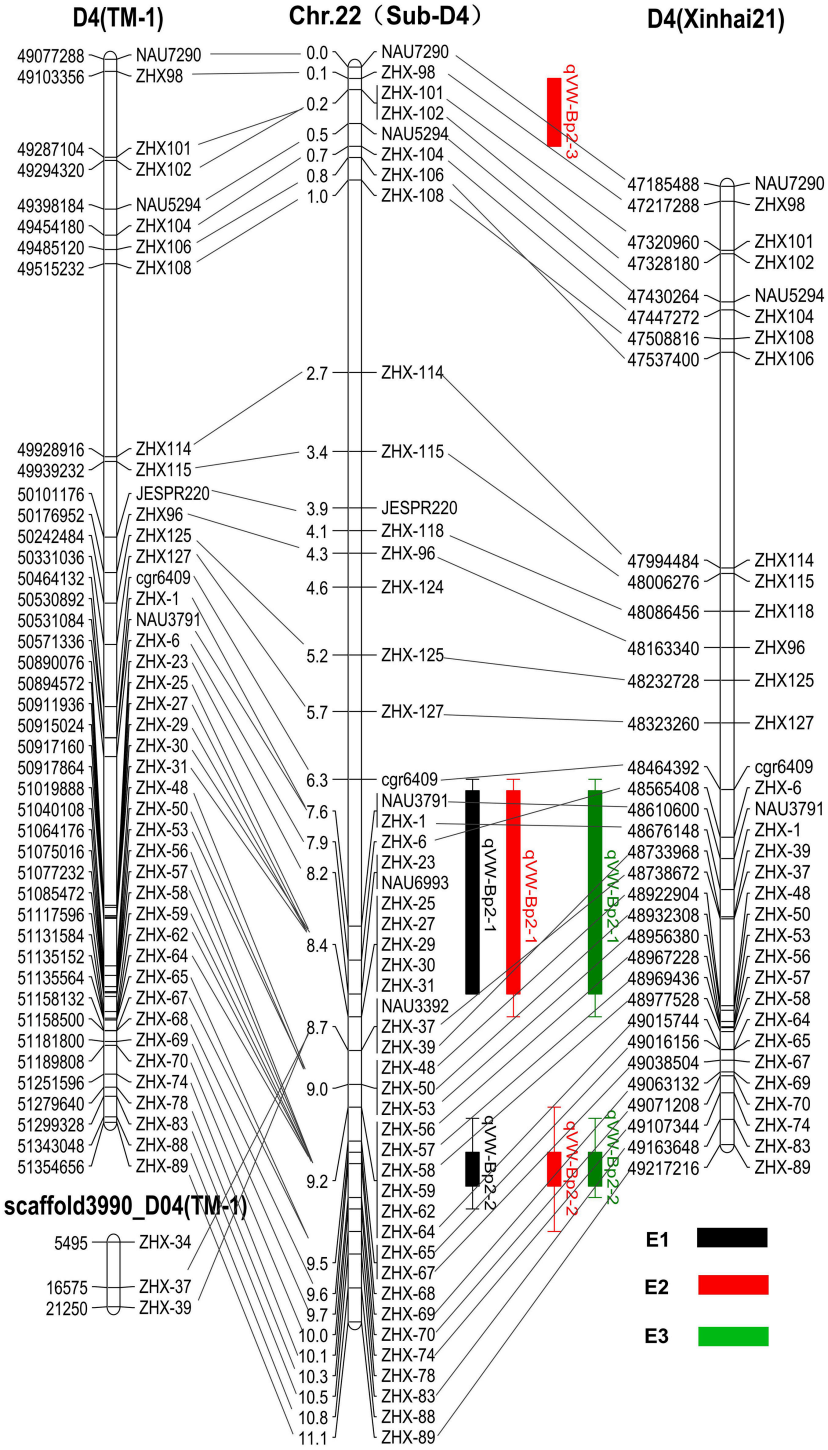
Comparison of genetic and physical maps of CSILSuVR043; mapping the major QTL for VW resistance. The middle map is the linkage group. The right and left maps are the physical maps based on the position of the sequence of markers on the linkage group against the complete genome sequence of *G. hirsutum* cv. TM-1 and *G. barbadense* cv. Xinhai 21. The lines connect the same marker locus on different maps. E1, E2, and E3 represent three VW resistance evaluation experiments in three different greenhouses.

Based on the available *G. hirsutum* (Zhang T. et al., 2015) and *G. barbadense* (Liu et al., 2015) genome sequences, 49 marker sequences were used for alignment analysis. First, we searched the sequence of 49 markers on the linkage group against the complete genome sequence of *G. hirsutum* cv. TM-1 and *G. barbadense* cv. Xinhai 21. The results showed the forward and reverse sequence of 34 markers were located in the *G. barbadense* cv Xinhai 21 genome, excluding markers JESPR220, ZHX-124, ZHX-27, ZHX-23, NAU6693, ZHX-25, ZHX-29, ZHX-30, ZHX-31, NAU3392, ZHX-59, ZHX-62, ZHX-68, ZHX-78, and ZHX-88 (Figure 4). The genetic orders of the 34 loci were consistent with their physical orders in the *G. barbadense* genome except for markers ZHX-6, ZHX-106, and ZHX-108. For *G. hirsutum* cv TM-1, the forward and reverse sequences of 49 markers were all located in the genome, and the genetic orders of these loci were consistent with their physical orders in the *G. hirsutum* cv TM-1 genome except for ZHX-37 and ZHX-39, which were located on scaffold3990_D04. The key markers cgr6409 and ZHX37 closely linked to the qVW-Bp2-1 locus, and ZHX57 and ZHX70 closely linked to the qVW-Bp2-2 locus in the local map, showed genetic and physical locations consistent with the *G. barbadense* genome. The precise physical distances between cgr6409-ZHX37 and ZHX57-ZHX70 in the *G. barbadense* genome were 254 and 140 kb, respectively. There were 36 and 20 putative genes in the qVW-Bp2-1 and qVW-Bp2-2 regions of the *G. barbadense* genome, respectively (Table 4), and there were 49 and 19 putative genes in the qVW-Bp2-1 and qVW-Bp2-2 regions of the *G. hirsutum* cv. TM-1 genome, respectively (Supplementary Table 6). Through comparing gene information of the qVW-Bp2-1 and qVW-Bp2-2 regions in the *G. barbadense* and *G. hirsutum* genomes, the results showed that six unique genes belonged to *G. barbadense* cv. Xinhai 21 in the Cgr6409-ZHX37 region. These are ORF17 (Chaperone protein dnaj-related), ORF18 (REF SRPP-like protein at1g67360), ORF19 (Cytochrome P450 78a5-like), ORF29 (Sodium transporter HTK1-like), ORF30 (Sodium transporter HTK1-like), and ORF36 (Histone H1). The open reading frame (ORF) lengths of the six genes were different, including ORF13 (Pre-mRNA-splicing factor ATP-dependent RNA helicase DHX16), ORF14 (Ran bp2 NZF zinc finger-like superfamily protein isoform partial), ORF16 (alpha-ketoglutarate-dependent dioxygenase alkB), ORF21 (Duf493 family protein), ORF25 (B-ZIP transcription isoform 2), and ORF34 (Transmembrane protein 214a). In the qVW-Bp2-2 region, the four unique genes in *G. barbadense* cv. Xinhai 21 included ORF3 (ZZ-type zinc finger-containing protein isoform 2), ORF7 (probable receptor-like protein kinase at1g67000-like), ORF10 (6-phosphogluconate decarboxylating 3), and ORF18 (Malectin receptor protein kinase family).

**Table 4:** Information on genes included in the qVW-Bp2-1 region flanked by the markers Cgr6409 and ZHX37 and the qVW-Bp2-2 region flanked by the markers ZHX57 and ZHX70 in *G. barbadense* L.cv.Xinhai 21.

	**Gene ID**	**Description**	**Length of ORF (bp)**	**Position**	***p*-value**
**qVW-Bp2-1(Cgr6409-ZHX37)**					
ORF1	GOBAR_DD30341	Proteasome subunit beta type-5	453	48459376-48461650	2.54E-47
ORF2	GOBAR_DD30342	Ribosome biogenesis gtpase	1,902	48462958-48467223	9.75E-171
ORF3	GOBAR_DD30343	60 s ribosomal protein l17-2	624	48468112-48470678	2.21E-102
ORF4	GOBAR_DD22682	Ankyrin repeat-containing protein at3g12360-like	2,424	48497380-48502027	0
ORF5	GOBAR_DD35297	Ankyrin repeat-containing protein at3g12360-like	861	48509548-48510660	3.62E-159
ORF6	GOBAR_DD22684	Ankyrin repeat-containing protein at3g12360-like	1,992	48512105-48516264	1.52E-138
ORF7	GOBAR_DD22685	Ankyrin repeat-containing protein at3g12360-like	687	48518172-48518977	3.17E-113
ORF8	GOBAR_DD35300	Ankyrin repeat-containing protein at3g12360-like	1,734	48524929-48526999	0
ORF9	GOBAR_DD22688	Ankyrin repeat-containing protein at3g12360-like	525	48539188-48539810	5.22E-72
ORF10	GOBAR_DD22689	Ankyrin repeat-containing protein at3g12360-like	627	48547338-48548409	4.37E-94
ORF11	GOBAR_DD22693	Ciliary neurotrophic factor	609	48569966-48570982	2.72E-104
ORF12	GOBAR_DD22694	Squamosa promoter-binding-like protein 2	1,413	48574845-48576853	0
ORF13	GOBAR_DD22695	Pre-mRNA-splicing factor ATP-dependent RNA helicase DHX16	2,322	48577846-48580167	0
ORF14	GOBAR_DD22697	Ran bp2 nzf zinc finger-like superfamily protein isoform partial	1,491	48584306-48590000	1.23E-173
ORF15	GOBAR_DD22700	Glucuronoxylan 4-o-methyltransferase 1-like	870	48604891-48605760	0
ORF16	GOBAR_DD22702	alpha-ketoglutarate-dependent dioxygenase alkb	1,158	48611845-48615440	0
ORF17	GOBAR_DD22703	Chaperone protein dnaj-related	297	48619239-48619535	4.90E-62
ORF18	GOBAR_DD22704	REF SRPP-like protein at1g67360	519	48623946-48626719	4.30E-117
ORF19	GOBAR_DD22705	Cytochrome p450 78a5-like	1,575	48635838-48639097	0
ORF20	GOBAR_DD22706	Flavonoid 3-methyltransferase-like isoform x1	726	48651110-48654144	1.95E-173
ORF21	GOBAR_DD22701	Duf493 family protein	597	48676130-48677850	5.88E-95
ORF22	GOBAR_DD22699	Non-specific lipid transfer protein gpi-anchored 1-like	582	48691074-48692628	2.57E-79
ORF23	GOBAR_DD22696	—NA—	201	48701954-48702154	
ORF24	GOBAR_DD35898	Retinol dehydrogenase 11	768	48730250-48733666	4.12E-74
ORF25	GOBAR_DD35897	b-zip transcription isoform 2	1,032	48736261-48741560	0.80
ORF26	GOBAR_DD35896	Hypothetical protein B456_012G180400	303	48743381-48743683	3.47E-17
ORF27	GOBAR_DD35895	Hypothetical protein B456_012G180400	345	48749870-48750214	6.37E-31
ORF28	GOBAR_DD35894	Hypothetical protein B456_012G180400	372	48752969-48753340	1.37E-52
ORF29	GOBAR_DD35893	Sodium transporter HTK1-like	1,101	48759294-48760394	0
ORF30	GOBAR_DD35892	Sodium transporter HTK1-like	771	48768255-48771199	2.52E-168
ORF31	GOBAR_DD35891	Hypothetical protein B456_012G180400	345	48773804-48774148	2.32E-72
ORF32	GOBAR_DD35464	Clathrin assembly protein at2g01600	1,869	48786666-48791242	0
ORF33	GOBAR_DD35465	C2 domain-containing family protein	618	48797176-48799073	1.87E-146
ORF34	GOBAR_DD35466	Transmembrane protein 214a	2,289	48801663-48809177	0
ORF35	GOBAR_DD35467	Nudix hydrolase 25	522	48813668-48816028	6.74E-122
ORF36	GOBAR_DD35468	Histone H1	2,688	48818599-48821974	0
**qVW-Bp2-2(ZHX57-ZHX70)**					
ORF1	GOBAR_DD29983	Clathrin assembly protein at2g25430	1,176	48967190-48968365	0
ORF2	GOBAR_DD29982	Probable RNA-dependent RNA polymerase 1	3,384	48971284-48977252	0
ORF3	GOBAR_DD29981	zz-type zinc finger-containing protein isoform 2	270	48985805-48986074	6.68E-51
ORF4	GOBAR_DD29980	Probable RNA-dependent RNA polymerase 1 isoform x1	3,681	48986741-48992222	0
ORF5	GOBAR_DD29979	zz-type zinc finger-containing protein isoform 4	1,044	48999858-49003565	1.71E-175
ORF6	GOBAR_DD30816	Hypothetical protein B456_012G184400	357	49027876-49028232	3.70E-64
ORF7	GOBAR_DD30817	probable receptor-like protein kinase at1g67000-like	2,994	49029729-49036596	0
ORF8	GOBAR_DD30818	Outer membrane usher papc	621	49041071-49042751	8.40E-106
ORF9	GOBAR_DD30819	Choline ethanolaminephosphotransferase 1	1,473	49047365-49055503	0
ORF10	GOBAR_DD30820	6-phosphogluconate decarboxylating 3	456	49055866-49056437	7.77E-68
ORF11	GOBAR_DD30821	Pentatricopeptide repeat-containing protein mitochondrial-like	1,473	49059446-49060918	0
ORF12	GOBAR_DD30822	UDP-glycosyltransferase 89a2-like	1,398	49065306-49066703	0
ORF13	GOBAR_DD30823	UDP-glycosyltransferase 89a2-like	1,269	49068319-49069746	0
ORF14	GOBAR_DD30823	UDP-glycosyltransferase 89a2-like	1,626	49072481-49074228	0
ORF15	GOBAR_DD30824	DNA glycosylase superfamily protein isoform 1	858	49079123-49082924	0
ORF16	GOBAR_DD30825	Cytochrome p450	1,542	49085314-49087354	0
ORF17	GOBAR_DD30826	Cytochrome p450	1,542	49089128-49092805	0
ORF18	GOBAR_DD30827	Malectin receptor protein kinase family	834	49094292-49096259	1.88E-40
ORF19	GOBAR_DD30828	Nucleolar protein 14	2,922	49097609-49103888	0
ORF20	GOBAR_DD30829	Truncated transcription factor cauliflower a-like	747	49106526-49109903	1.03E-168

### Expression analysis of candidate genes

**Figure 5:**
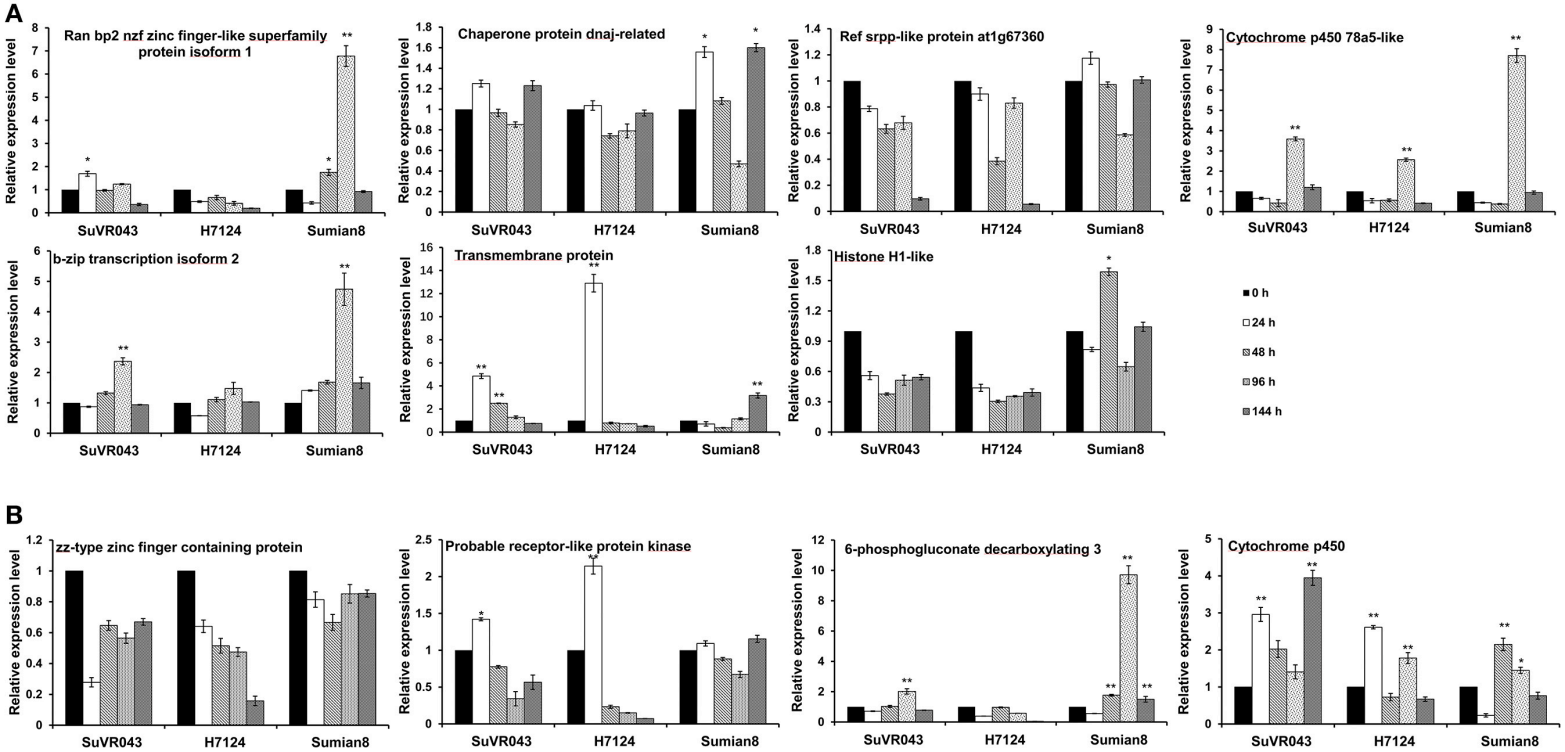
Expression analysis of candidate genes in the qVW-Bp2-1 **(A)** and qVW-Bp2-2 **(B)** regions. ^*^Indicates significance at *P* < 0.05, ^**^Indicates significance at *P* < 0.01.

For analysis of the candidate VW resistance genes in the qVW-Bp2-1 and qVW-Bp2-2 regions, 11 genes were selected based on previous reports, sequence difference and gene ontology analysis, and analyzed their expression profiles by qRT-PCR using root tissue with *V. dahliae* treatment at different stages of CSIL SuVR043, *G. barbadense* cv. H7124 and *G. hirsutum* cv. Sumian 8. In the qVW-Bp2-1 region, the expression pattern of Cytochrome P450 78a5-like was similar, and the expression levels increased 3.6, 2.6, and 7.7-fold at 96 h after inoculation and then decreased rapidly in CSIL SuVR043, H7124 and Sumian 8, respectively. Transmembrane protein 214a expression began to increase by 4.4 and 13.1-fold and reached the maximum value at 24 h and decreased rapidly at 48 h after inoculation in CSIL SuVR043 and H7124, however, only increase just by 2.3-fold at 144 h after inoculation in Sumian 8. The expression levels of two genes, B-Zip transcription 2 and Ran bp2 NZF zinc isoform finger-like superfamily protein isoform 1 were changed in response to *V. dahliae* only in CSIL SuVR043 and Sumian 8, but were not changed in H7124. The expression levels of Histone H1-like were changed only in Sumian 8, and the expression levels of Ref srpp-like protein at1g67360 were not changed in response to *V. dahliae* among the three cotton varieties (Figure 5A). In the qVW-Bp2-2 region, Cytochrome P450 expression began to rise at 24 h after inoculation, decreased at 48 h, and then reached a maximum at 96 h in CSIL SuVR043 and H7124. In Sumian 8, the expression of Cytochrome P450 began to increase at 48 h, and the up- regulation time was shorter than that of CSIL SuVR043 and H7124. The expression level of Probable receptor-like protein kinase was increased by 1.4- and 2.3-fold at 24 h after inoculation in CSIL SuVR043 and H7124, but was not changed in Sumian 8. The expression levels of 6-phosphogluconate decarboxylating 3 were changed in response to *V. dahliae* only in CSIL SuVR043 and Sumian 8 and were not changed in H7124. The expression levels of ZZ-type zinc finger–containing protein were not changed in response to *V. dahliae* among the three cotton varieties (Figure 5B). These results showed that Cytochrome P450, Transmembrane protein 214a and probable receptor-like protein kinase may play important roles in CSIL SuVR043 resistance to VW.

### Functional analysis of candidate genes by VIGS

**Figure 6:**
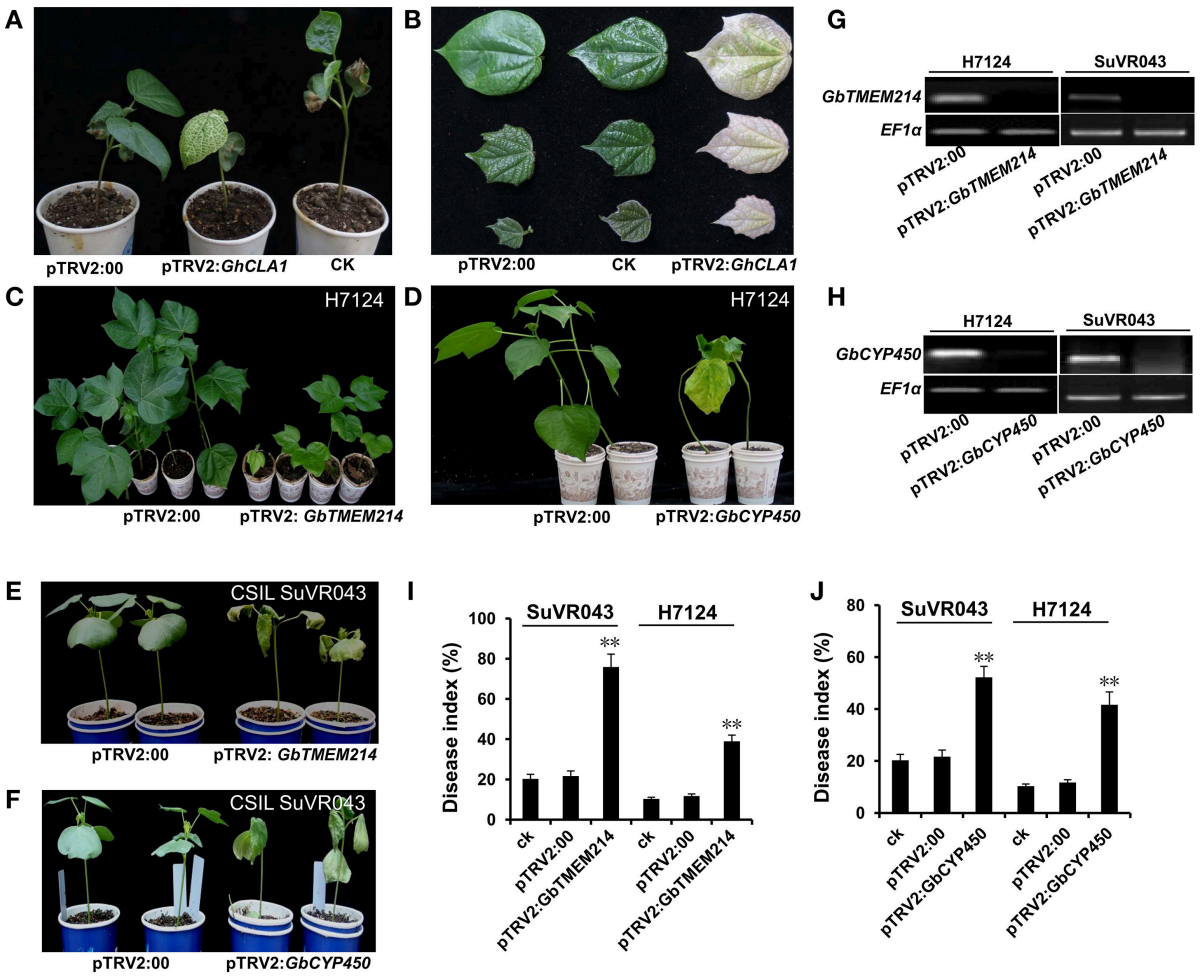
Resistance function analysis of the *GbCYP450* and *GbTMEM214* genes by VIGS. **(A)** The phenotype of the cotton plants 14 days after VIGS. The untreated cotton was used as the CK. The empty vector pTRV2:00 was used as a negative control. The cotton gene *GhCLA1* was used as a positive control; VIGS of *GhCLA1* results in a phenotype of white leaves. **(B)** The phenotypes of the cotton leaves 14, 21, and 28 d after VIGS with the empty vector pTRV2:00, no treatment, and VIGS with the vector pTRV2-*GhCLA1*. **(C,D)** The phenotypes of H7124 under infection by *V. dahliae* isolate BP2 after VIGS with *Agrobacterium* carrying pTRV2:GbTMEM214, pTRV2:GbCYP450 and pTRV2:00. The photos were taken at 42 days after *V. dahliae* inoculation. **(E,F)** The phenotypes of CSIL SuVR043 under infection by *V. dahliae* isolate BP2 after VIGS with *Agrobacterium* carrying pTRV2:GbTMEM214, pTRV2:GbCYP450, and pTRV2:00. Photos were taken at 42 d after *V. dahliae* inoculation. **(G,H)** RT-PCR analysis of the expression levels of the genes *GbTMEM214* and *GbCYP450* in the silenced lines. **(I,J)** The disease index of plants with silenced *GbTMEM214* and *GbCYP450* genes. The results were evaluated at 28 d after *V. dahliae* inoculation, with three replications containing at least 20 plants each. Asterisks indicate statistically significant differences, as determined by Student's *t*-test (^**^p < 0.01).

To further reduce the range of candidate genes in the introgressed *G. barbadense* chromosome segment, we applied VIGS to analyze the roles of Cytochrome P450 (*GbCYP450*), Transmembrane protein 214a (*GbTMEM214*) and probable receptor-like protein kinase (*GbRLK*) in resistance to VW based on the results of expression analysis. *V. dahliae* isolate Bp2 was inoculated into *GbCYP450*-deficient, *GbTMEM214*-deficient, *GbRLK*-deficient cotton lines. The results of the RT-PCR analysis showed that the expression levels of *GbCYP450, GbTMEM214* and *GbRLK* decreased significantly in seedlings 2 weeks after VIGS infiltration in H7124 and CSIL SuVR043. The disease indices were calculated after 4 weeks of inoculation. For the gene *GbCYP450*, the disease index of H7124 and CSIL SuVR043 with *GbCYP450* deficiency reached 41.7 and 53.8% after VIGS infiltration compared to 10.3% (H7124, control plant), 11.7% (H7124, pTRV2:00), 20.3% (CSIL SuVR043, control plant), and 21.7% (CSIL SuVR043, pTRV2:00). For *GbTMEM214*, the disease indices of H7124 and CSIL SuVR043 with *GbTMEM214* deficiency reached 38.9 and 75.9% after VIGS infiltration. For *GbRLK*, the disease indices of H7124 and CSIL SuVR043 with *GbRLK*-deficient were 13.3 and 27.1% after VIGS infiltration, and no significant differences were noted compared to the control (data not shown). The results showed that H7124 and CSIL SuVR043 with silenced *GbCYP450* and *GbTMEM214* had decreased resistance to VW. This indicates that *GbTMEM214* and *GbCYP450* in this study played important roles in CSIL SuVR043 resistance to VW and are candidate genes in the qVW-Bp2-1 and qVW-Bp2-2 regions, respectively (Figure 6).

## Discussion

Many *G. barbadense* genotypes have been known to carry high levels of resistance to VW (Wilhelm et al., 1974; Zhang J. F. et al., 2012; Zhou et al., 2014), In particular, *G. barbadense* cv. H7124 is grown extensively and is a widely used VW-resistant cultivar in China for analysis of the inheritance of resistance, mapping of resistance QTL, cloning of resistance genes and breeding. To date, many VW resistance QTLs have been detected on *G. barbadense* cv. H7124 chromosomes (Yang et al., 2008; Wang et al., 2014), but its resistance has not been successfully transferred into commercial upland cotton due to the linkage drag. In this study, we developed a *G. barbadense* cv. H7124 CSIL SuVR043 with higher resistance to VW than the recurrent parents through the multiple cycles of backcross and disease resistance identification, and there were no significant differences between CSIL SuVR043 and the recurrent parents in agronomic characters including fiber quality and yield. This indicated that the chromosome fragments of CSIL SuVR043 from *G. barbadense* cv. H7124 could significantly improve the resistance of upland cotton to VW and that CSIL SuVR043 could be as an ideal material for breeding the the resistance benefits of introgression from *G. barbadense* into upland cotton.

The identification of VW resistance QTLs is difficult. To date, a total of 306 disease resistance QTLs have been reported, and 193 QTLs are associated with VW resistance (Zhang J. F. et al., 2015). Most VW resistance QTLs could not be detected repeatedly in different environmental conditions or repeated experiments. There are a number of factors that contribute to this phenomenon. First, most VW resistance QTLs have low contributions to VW resistance (Zhang J. F. et al., 2015; Palanga et al., 2017). To increase the marker density of the genetic map when mapping QTLs, the varieties with the greater genetic background difference were selected to be the parents. This minimized the contribution values due to the background genetic effects. Furthermore, an artificial grading system for resistance evaluation makes it very difficult to quantitatively and accurately evaluate the phenotypic features of plants responses to VW; any experimental errors in VW resistance screening may lead to failure to detect the QTLs for VW resistance (Zhang J. F. et al., 2015). Second, VW infections are highly sensitive to environmental factors. Most VW resistance QTLs have specialization to the different *V. dahliae* isolates, and the distinct gene(s) control the resistance to the different *V. dahliae* isolates. Many mapping of VW resistance QTL used the different *V. dahliae* isolates or evaluated the phenotype of resistance in different environment (Yang et al., 2008; Wang et al., 2014). The pathogenicity and sporulation ability of the same *V. dahliae* isolate were different in the different culture conditions, especially for *V. dahliae* isolates present in the field, *V. dahliae* is a mixture of many strains and the fungal community structure is constantly changed in response to the changes in the environment (Xia et al., 1998). It is possible to obtain different results for disease resistance QTLs identification in different environments or using different strains. Thirdly, the low genome coverage of molecular markers in many mapping studies has limited the detection of QTLs genome-wide at a high resolution (Zhang J. F. et al., 2015; Palanga et al., 2017).

In this study, qVW-Bp2-1 and qVW-Bp2-2 were both detected stably in three independently repeated experiments, and the average explained phenotypic variances and position of qVW-Bp2-1 and qVW-Bp2-2 (15.98–16.51% for qVW-Bp2-1 and 21.06–24.61% for qVW-Bp2-2) were relatively fixed. This means that these two QTLs for VW resistance were accurate and stable. The development of CSIL SuVR043 was the key for the QTL identification in this study. Due to CSIL SuVR043 contained one substituted segments from the donor *G. barbadense* cv. H7124, all the genetic background variation between CSIL SuVR043 and G. hirsutum cv. Sumian 8 is associated with the substituted segment. This circumstance minimizes background genetic effects and allows more reliable QTL detection and evaluation of high contribution values (Zamir, 2001; Wang et al., 2008, 2014). Meanwhile, Precautions were needed to increase the accuracy of our data and thus to reduce experimental errors. We performed the three independently repeated experiments in three different greenhouses at the same time and inoculated the plants using the same batch of *V. dahliae*. In every experiment the parents and F_2:3_ family lines were planted in a completely randomized design with three replications. For each replicate, the disease grades of at least 15 plants were counted for every F_2:3_ family line in the three independent repeated experiments. The high heritability (0.64–0.72) in the resistance investigation (Figure 3) suggested that the phenotypic variability of three independently repeated experiments was not subject to environmental effects. For increasing the density of the genetic map, we developed 189 SSR primer pairs based on the *G. raimondii* genome sequence (32304099–35343399), and used an F_2_ population (1,100 individuals) to construct a linkage group which containing 49 SSRs and 32 loci, spanning 11.1 cM and covering the whole substituted segment. The average genetic distance between loci was just 0.35 cM. The construction of the high-density genetic map circumvented the problem of low genome coverage of the genetic map, and was another key factor for detection of two loci in the substituted segment,

In the past, some researchers have also detected VW resistance QTLs in Chr 22 (D04). To date, at least nine resistance QTLs on Chr 22 (D04) have been reported; Additionally, one resistance QTL cluster linked with markers NAU2235, BNL3873, CIR218, CIR122, and NAU2291 were identified by meta-analysis of VW (Zhang J. F. et al., 2015). Further comparing the sequences of markers NAU2235, BNL3873, CIR218, CIR122, and NAU2291 against the complete genome sequence of *G. raimondii*, we found that the markers BNL3873, CIR218, CIR122, and NAU2291 were located on the introgressed chromosome segment or were neighboring it (Supplementary Table 7). Palanga et al. also identified 3 resistance QTL clusters explaining 3.7–8.6% on Chromosome 22 (D04) (Palanga et al., 2017), indicating that there was a resistance-related QTL in the target region of this study. Yang et al. (2008) used *G. barbadense* cv. H7124 and *G. hirsutum* cv. Junmian 1 as material to construct an F_2_ population and mapped a VW resistance QTL, which explained 20.3% of the phenotypic variation, to the region NAU3437-NAU3392 of Chr. 22 for the non-defoliating *V. dahliae* isolate Bp2,. Wang et al. (2014) used the interspecific CSILs to map a VW resistance QTL linked to the marker JESPR220 on Chromosome 22 (D04) under greenhouse conditions; the QTL explained 2.2% of the phenotypic variation. In this study, we used CSIL SuVR043 with its published resistance QTL region to construct F_2_ population plants and detected two major resistance QTLs (qVW-Bp2-1 and qVW-Bp2-2) for the nondefoliating *V. dahliae* isolate Bp2 on the introgressed chromosome segment. The key marker NAU3392 was located on the qVW-Bp2-1 region between the markers cgr6409 and ZHX37 in our genetic map. The region qVW-Bp2-2 was adjacent to qVW-Bp2-1, and the distance between them was just 1.5 cM. Therefore, we speculated that qVW-Bp2-1 and qVW-Bp2-2 may be the same resistance locus detected by Yang et al. (2008). Due to the large population and increased number of linked markers in this study, the resistance QTL locus detected by Yang et al. (2008) was separated into two loci in our results. In this study, the qVW-Bp2-1 and qVW-Bp2-2 regions contained 14 and 11 markers, respectively; most of these markers were co-separated and restricted positioning was reduced. Therefore, we expected to further narrow the field of resistance loci by expanding the population.

*V. dahliae* of cotton is divided mainly into defoliating and nondefoliating isolates, and each isolate also includes many races. Significant differences in pathogenicity exist among different races (Short et al., 2015); therefore, breeding broad-spectrum disease resistant cotton varieties will be the main solution to the cotton production problems caused by *V. dahliae*. To date, some scholars have detected some broad-spectrum disease resistance loci (Ning et al., 2013; Wang et al., 2014), but most resistant QTLs are specific to certain races. Therefore, it is probably an appropriate choice to breed broad-spectrum resistant cotton by polymerization of the different pathogen resistance QTLs through marker-assisted selection. In this study, resistance loci were detected for the nondefoliating isolate Bp2, but it was not determined whether these resistance loci are effective against other strains.

In recent decades, many genes related to cotton VW resistance have been cloned, but these were obtained by analyzing differences in transcript profiles or by homologous cloning (Xu et al., 2011, 2014; Zhang B. L. et al., 2012). In this study, we identified three candidate genes by analyzing the sequence and expression of genes in two QTL regions following QTL mapping. Those genes encoded *GbTMEM214, GbCYP450*, and *GbRLK*, and their expression levels in CSIL SuVR043 and *G*. *barbadense* cv. H7124 were significantly different from those in *G. hirsutum* cv. Sumian 8. The results of functional analysis of the three candidate genes by VIGS showed that resistance to VW of CSIL SuVR043 and *G*. *barbadense* cv. H7124 was significantly reduced after silencing *GbTMEM214* and *GbCYP450*, but not *GbRLK*. This result suggests that *GbRLK* might not be a key gene in the resistance pathways or may be complemented by other genes.

The transmembrane protein (TMEM) family has been known to be involved in many physiological processes, such as intercellular and intracellular signal transduction, related disease immunity, tumor occurrence and development, ion channels, mediating cell chemotaxis, adhesion, apoptosis, autophagy, and more (Katoh and Katoh, 2004; Miller, 2006; Gregersen et al., 2014; Lees et al., 2017). However, there have been few reports on the function of *TMEM* family members in plants, indicating that their study in plants is still in its infancy. Previous studies have shown that the *TMEM214* gene was involved in the process of apoptosis in human cells and in signal transduction of cancer cells (Li C. et al., 2013). In this study, the ORF length of *GbTMEM214* gene was different in the released genome sequences of *G. hirsutum* cv. TM-1 and *G. barbadense* cv. Xinhai21 (Supplementary Table 8). The results of location, expression and VIGS analysis suggested that the *GbTMEM214* gene might play a key role in the VW resistance process in cotton. Then, it is possible to provide some perspective for the future research on the function of the *TMEM* gene family in plants and a new candidate gene for VW resistance research in cotton. CYP450, widely present in microbes, plants and animals is a large protein family and is divided into many subfamilies. According to existing reports, the CYP450 protein is involved in the synthesis of a variety of substances, including not only lignin, suberin, keratin and other macromolecules but also hormones, signal molecules and other small molecules (Werckreichhart et al., 2001). Some *CYP450* genes also play important roles in plant disease resistance (Smigocki and Wilson, 2004; Wang et al., 2004; Li W. Q. et al., 2013; Sun et al., 2014; Xu et al., 2014; Yang L. et al., 2015). In this study, *GbCYP450* belongs to the *CYP72A* subfamily by analysis of a phylogenetic analysis of CYP450 proteins in *Arabidopsis* using the software MEGA5.0, and the copy number of *GbCYP450* gene was different based on the released genome sequences of *G. hirsutum* cv. TM-1 and *G. barbadense* cv. Xinhai21 (Supplementary Table 8). Meanwhile, the expression and VIGS analysis also showed that *GbCYP450* also plays an important role in VW resistance.

## Conclusion

In this study, CSIL SuVR043 containing a single and homozygous chromosome introgressed segment of *G. barbadense* cv. H7124 Chr 22 (D04) was used to construct an F_2_ population for mapping of VW resistance QTL. We constructed a linkage group with 32 loci and an average of 0.35 cM genetic distance between loci using an F_2_ population containing 1,100 individuals. Two major resistance QTLs for *V. dahliae* isolate Bp2, qVW-Bp2-1, and qVW-Bp2-2, were detected in three independently repeated experiments, which explained an average of 16.38 and 22.36%, respectively, of the observed phenotypic variation. The sequence, gene expression and VIGS analysis revealed that two candidate genes, *GbCYP450* and *GbTMEM214*, might play the key roles in the VW resistance for SuVR043. Hence, these results provide solid foundation for fine mapping and cloning of resistance genes on the substituted segment in the future.

## Author contributions

JZ and SX: designed the experiments and wrote the manuscript; JZ: collected the genotype and phenotype data; JL and QW: collected the phenotype data; JX: collected the genotype data; LZ: analyzed the data.

### Conflict of interest statement

The authors declare that the research was conducted in the absence of any commercial or financial relationships that could be construed as a potential conflict of interest.
