# Supplementary figures and images for: Quantitative Trait Locus Mapping and Candidate Gene Analysis for Verticillium Wilt Resistance Using Gossypium barbadense Chromosomal Segment Introgressed Line
Source: Front Plant Sci. 2018 May 30;9:682. doi: 10.3389/fpls.2018.00682 (PMC5988901; doi:10.3389/fpls.2018.00682)

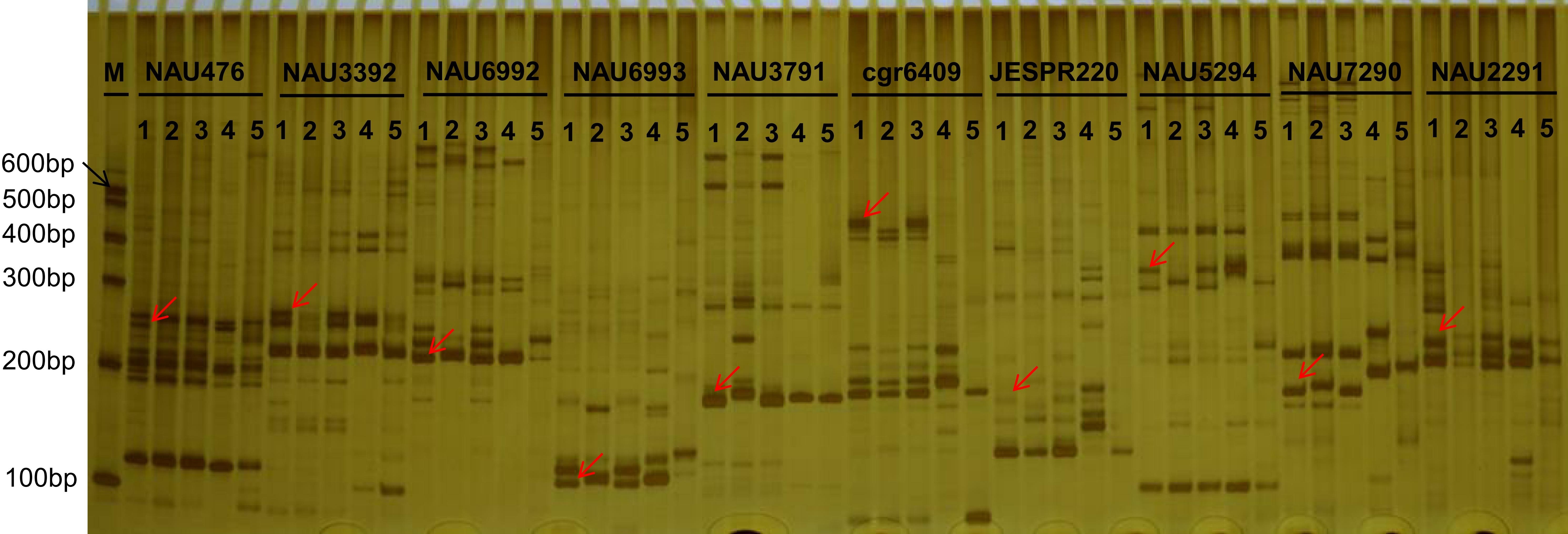

Supplement: Supplementary file 9 [file Image_1.JPEG]
