# Supplementary material for: Quantitative Trait Locus Mapping and Candidate Gene Analysis for Verticillium Wilt Resistance Using Gossypium barbadense Chromosomal Segment Introgressed Line
Source: Front Plant Sci. 2018 May 30;9:682. doi: 10.3389/fpls.2018.00682 (PMC5988901; doi:10.3389/fpls.2018.00682)
Supplement: Supplementary file 10 [file Presentation_1.PDF]

Supplementary Material

Quantitative trait loci mapping and candidate gene analysis for *Verticillium* wilt resistance using *Gossypium barbadense* chromosomal segment introgressed line

Jun Zhao, Jianguang Liu, Jianwen Xu, Liang Zhao, Qiaojuan Wu, Songhua Xiao*

*** Correspondence:** Dr. Songhua Xiao, E-mail: njxsh@sina.com.

Supplementary Figures and Tables

Supplementary Figure 1. Polymorphic analysis of ten markers in different cotton varieties.

M: marker; lane 1-5 represent *G. barbadense* L. cv. H 7124, *G. hirsutum* L. cv. Sumian 8, CSIL SuVR043, *G. raimondii* and *G. herbaceum* race *africanum*, respectively; The red arrow showed the different and dominant band in *G. barbadense* L. cv. H7124.

Supplementary Table 1. The primers for the quantitative RT-PCR.

Supplementary Table 2. The primer pairs sequence with restriction site for the construction of VIGS vectors.

Supplementary Table 3. The information of the SSR primers in the D04 (Chr 22) group of *G. hirsutum*×*G. barbadense* maps (Zhao et al., 2012).

Supplementary Table 4. The information of the primers located in the introgressed chromosome segment from D04 (Chr.22) cotton chromosomes based on *G. hirsutum*×*G. barbadense* maps.

Supplementary Table 5. The information of the development SSR primers.

Supplementary Table 6. The information of genes including the qVW-Bp2-1 region flanked by marker Cgr6409 and ZHX37, and the qVW-Bp2-2 region flanked by marker ZHX57 and ZHX70 in *G.hirsutum* L.acc.TM-1.

Supplementary Table 7. The information of the primers linked with the resistance QTL clusters and hotspots.

Supplementary Table 8. The sequences of the candidate genes.
